# Cannabis consumers’ preferences for legal and illegal cannabis: evidence from a discrete choice experiment

**DOI:** 10.1186/s12889-024-19640-1

**Published:** 2024-09-04

**Authors:** Jin Xing, Yuyan Shi

**Affiliations:** Herbert Wertheim School of Public Health and Human Longevity Science, University of California San Diego, 9500 Gilman Drive, La Jolla, CA 92093-0628 USA

**Keywords:** Cannabis, Illegal markets, Cannabis legalization, Cannabis dispensary, Discrete choice experiment, Mixed logit

## Abstract

**Background:**

In U.S. states that legalized and commercialized recreational cannabis, cannabis sales in illegal markets are still sizable or even larger than those in legal markets. This study aimed to assess cannabis consumers’ preferences for purchasing cannabis from legal and illegal markets and estimate the trade-offs under various policy scenarios.

**Methods:**

963 adults were recruited, who used cannabis in the past year and lived in a state with recreational cannabis legalization. In a discrete choice experiment, participants chose purchasing cannabis from a legal dispensary or an illegal dealer with varying levels in product attributes including quality, safety, accessibility, potency, and price. Mixed logit models were used to analyze preferences.

**Results:**

The likelihood of choosing legal cannabis increased with a higher quality, the presence of lab test, a shorter distance to seller, a higher tetrahydrocannabinol level, and a lower price. The likelihood of choosing illegal cannabis increased with a higher quality, a shorter distance to seller, and a lower price. Among product attributes, quality and accessibility were perceived to be the most important for legal cannabis and price was perceived to be the most important for illegal cannabis. Policy simulations predicted that improving quality, ensuring safety, allowing delivery services, increasing dispensary density, and lowering prices/taxes of legal cannabis may reduce illegal cannabis market share.

**Conclusions:**

In the U.S., cannabis consumers’ preferences for illegal cannabis were associated with both legal and illegal cannabis product attributes. Policies regulating legal cannabis markets should consider potential spillover effects to illegal markets.

**Supplementary Information:**

The online version contains supplementary material available at 10.1186/s12889-024-19640-1.

## Introduction

A key argument for recreational cannabis legalization is that fostering open and competitive legal markets has potential to reduce the size of illegal markets and eventually eliminate them. [1] Cannabis sales in both legal and illegal markets may have adverse public health, social, and economic consequences. Because illegal markets are not regulated, however, there are more concerns on their product safety than legal markets, such as contamination with harmful chemicals, additives, and other drugs, and unknown or very high level of potency that increases the risk of overdose. Interaction with illegal dealers also increases the chance of being offered and initiating the use of other more dangerous illegal drugs. [2] Illegal cultivation often involves crimes, labor exploitation, and environmental damages. [3–5] State governments also lose billions of tax revenues from illegal cannabis sales. [6] In the U.S. and Canada, after legalization, the legal cannabis markets have expanded dramatically. [7–10] Nonetheless, cannabis sales in illegal markets are still sizable or even larger than those in legal markets. For example, illegal cannabis sales in California reached $8 billion annually, twice as large as legal sales, in 2021 after three years of cannabis commercialization. [11] The assumption that cannabis consumers would switch from illegal markets to legal markets seems lacking data support after years of recreational cannabis legalization and legal market expansion. Understanding the causes of demand for illegal cannabis and implementing regulatory strategies to divert demand from illegal to legal markets are urgent tasks for cannabis policymaking.

Little research has examined cannabis consumers’ choices between legal and illegal cannabis, mainly because data on legal and illegal market transactions at individual level are often not concurrently available to researchers. The only two studies by Amlung et al. conducted hypothetical purchase tasks among U.S. and Canadian cannabis consumers to estimate the responsiveness of cannabis demand for legal and illegal sources to prices of the products from the two sources. [12, 13] They found that a greater price of cannabis from one source motivated substitution with cannabis from the other source. Such substitution was asymmetric: cannabis consumers attached a greater preference to legal cannabis than illegal cannabis. Price was the only factor assessed in these studies.

Lower prices in illegal cannabis markets have been a common explanation for illegal sales continuing to surpass legal sales. [14] Costs of legal cannabis include licensing fees, excise and sales taxes, rent for a storefront, advertising expenses, and non-monetary efforts such as application for licenses, staff training, and following various regulatory requirements, all of which are not paid by illegal sellers. It is plausible to assume that prices of legal cannabis are higher than those of illegal cannabis if these additional costs are at least partially passed on to consumers. Canada data after recreational cannabis legalization seem to support this assumption. [15]

Price, however, is not the only factor influencing cannabis consumers’ choices between legal and illegal cannabis. [16–18] For example, cannabis sold in legal markets presumably has a better quality because licensed cultivators need to meet certain cultivation conditions and follow safety regulations and cultivation procedures. [19] Legal products are required to do lab tests for cannabinoids, terpenes, and contaminations from chemicals, pesticides, heavy metals, etc., so product safety is to a large extent guaranteed. Labels are also required on legal cannabis packages to inform potency levels for tetrahydrocannabinol (THC) and cannabidiol. In contrast, illegal cannabis does not need to comply with these requirements. One Canadian report found that illegal cannabis was more contaminated and inaccurately labeled than legal cannabis. [20] Consumers hence likely prefer legal cannabis to illegal cannabis because of these factors when prices are equal. Further, in the U.S. local jurisdictions opt in or out for dispensary licensing, which introduces huge variations in accessibility of legal cannabis. In cities that do not license dispensaries or have strict zoning or density ordinances may make legal cannabis hard to access and unintendedly leave room for illegal markets. The list of factors explaining demand for legal and illegal cannabis can continue. Empirical research is needed to estimate the potential impacts of policies regulating these factors.

This study aimed to assess cannabis consumers’ preferences for purchasing cannabis from legal and illegal markets and estimate the trade-offs under various hypothetical policy scenarios. We adopted discrete choice experiment (DCE), a common approach in tobacco research and health economics. [21, 22] In a DCE, participants are asked to choose between hypothetical alternatives described by a series of attributes with varying levels. There are several advantages of DCEs relative to observational data from population surveys or sales transactions. DCEs directly elicit participants’ choices between different alternatives, so data on individual choices between legal and illegal cannabis can be made available to researchers. Because choices are hypothetical, DCEs provide opportunities to examine choices that do not yet exist in reality, so the impacts of a potential policy could be estimated. DCEs usually yield stronger causal inferences because they control choice attributes and associated levels and estimate within-individual variations. In this study, we focused on five cannabis attributes including quality, safety, accessibility, potency, and price. We assessed users’ preferences in general cannabis consumer population as well as subgroups categorized by cannabis use purposes and frequency. To make the findings more relevant and useful to policymaking, we also performed policy simulations to estimate market shares of legal and illegal cannabis under different policy scenarios.

## Methods

### Data collection and study sample

Data were collected online in May 2019. Participants were recruited through Qualtrics from various online panels. The inclusion criteria included (1) being 21 years or older so cannabis purchase is legal, (2) reporting cannabis use in the past 12 months, and (3) living in one of the eight U.S. states with recreational cannabis legalization (California, Colorado, Washington, Oregon, Nevada, Massachusetts, Maine, and Michigan) at the time of data collection. To make our sample representative of U.S. cannabis consumer population, we assigned sampling quotas to subgroups by key socio-demographic characteristics (age, sex, and education) to match the sample characteristics in the 2019 National Survey on Drug Use and Health (NSDUH), a large national, probability-based survey in the U.S. [23] The final sample comprised 963 cannabis consumers.

This study was approved by Institutional Review Board at University of California San Diego.

### Discrete choice experiment design

Our DCE design followed the best practices recommended by the Task Force. [24] Detailed experiment instructions and a choice scenario example can be found in Survey S1.

In each choice scenario, participants selected from two cannabis flower alternatives: (1) legal cannabis sold by a licensed dispensary, and (2) illegal cannabis sold by an illegal dealer, who could be strangers, friends, or relatives. Opt-out option was also available. Attributes and associated levels for each alternative are provided in Table 1. Figure S1 shows a choice scenario example.

**Table 1:** DCE attributes and Associated levels

Attribute	Legal Status of Purchase Source	
	Legal Dispensary	Illegal Dealer
**A. Quality**		
	A1. Low	A1. Low
	A2. Medium	A2. Medium
	A3. High	A3. High
**B. Safety (Lab Test)**		
	B1. Yes	
	B2. No	B2. No
**C. Accessibility (Distance between Seller and Home)**		
	C1. Deliverable	C1. Deliverable
	C2. 1 mile	C2. 1 mile
	C3. 10 miles	C3. 10 miles
	C4: 50 miles	C4: 50 miles
**D. Potency (THC Level)**		
	D1. Low: 10%	
	D2. Medium: 20%	
	D3. High: 30%	
	D4. Unknown	D4. Unknown
**E. Price (per 1/8 Ounce Flower)**		
	E1. $20	E1. $20
	E2. $30	E2. $30
	E3. $40	E3. $40
	E4. $50	E4. $50

*Notes*: THC stands for tetrahydrocannabinol

We selected attributes that are important to cannabis consumers. A systematic review suggested that the most common factors influencing cannabis purchase included price, quality, recommendations from sellers and acquaintances, route of administration, and packaging. [17] An experimental study identified quality, strain, price, THC level, and pesticide contamination as the most important factors. [18] A large population survey indicated that quality, safety, accessibility, and price were the most important factors. [25] Because cognitive burden increases with the number of attributes, [24] in this study we focused on five attributes: (1) quality, (2) safety (presence of lab test), (3) accessibility (proximity to seller), (4) potency (THC level), and (5) price. These attributes are also modifiable by policies and hence highly relevant to policymaking. Legal cannabis was described by all the five attributes. Illegal cannabis was described by quality, accessibility, and THC level. Lab test was defined to be absent and THC level was defined to be unknown for all the illegal cannabis choices because lab tests and potency labels are not required for cannabis sold in illegal markets.

We then selected attribute levels based on market data or literature. The three levels of quality were low, medium, and high. Definitions of quality in literature varied and were often vague, [17] hence we defined quality by visual appeal and provided detailed text and visual descriptions (Technical Note S1). The two levels associated with safety were presence and absence of lab test. The four levels of accessibility to sellers were deliverable, 1 mile, 10 miles, and 50 miles from home. THC and price levels were based on product menu on Weedmaps, the popular online platform for cannabis dispensaries. The four levels of THC were low (10%), medium (20%), high (30%), and unknown. The four levels of price for 1/8 ounce cannabis flower, the most common selling weight in the U.S., were $20, $30, $40, and $50.

We randomly selected 81 scenarios and partitioned them into nine blocks, each of which contained nine choice scenarios. [26]. Participants were randomly assigned with one of the nine blocks and responded to all the nine choice scenarios in that block. Our DCE design achieved an 85.2% D-efficiency.

To enhance DCE data quality, before starting the DCE participants were presented with detailed textual and visual descriptions of attributes and levels and provided with an example DCE scenario to practice (Survey S1). We noted that some factors were not described by the five attributes and should be assumed to be constant in all the choices (Figure S2). We included an attention check asking for the day of the week, which were correctly answered by all the participants.

### Sample demographic and behavioral characteristics

Along with DCE questions, in the survey we also collected participants’ demographic and behavioral data, including age, sex, education, race/ethnicity, cannabis use purposes and frequency (Survey S1). Cannabis use purposes had three categories: medical only (past-year use primarily for medical purposes to treat health conditions or mitigate symptoms), recreational only (past-year use primarily for recreational purposes to attain pleasure or satisfaction), and dual purposes (past-year use for both medical and recreational purposes). Cannabis use frequency had two categories: occasional use (use < 20 days in the past 30 days) and regular use (use ≥ 20 days in the past 30 days).

### Statistical analysis

We used mixed logit (or random-parameters logit) model to examine associations between preferences for legal and illegal cannabis and product attributes. It assumes that the probability of choosing an alternative is a function of attribute levels and a random error accounting for individual-specific variations in preferences. [27] It overcomes pitfalls of the conventional conditional logit (or multinomial logit) model by allowing (1) relaxation of the irrelevant alternative independence assumption, (2) heterogeneities of preference coefficients across individuals, and (3) flexible substitution patterns between alternatives. [28] In our study the dependent variable represented the likelihood of choosing one alternative over others and the covariates included attribute levels as well as alternative-specific constants that captured baseline preferences for legal and illegal cannabis relative to opt-out. All the attributes were treated as categorical variables except for price, which was modeled as a continuous variable. The coefficient was assumed to follow an independent normal distribution. The coefficients were modeled separately for legal and illegal cannabis because cannabis consumers may attach different preferences to the same attribute level. Standard errors were clustered at individual level.

We calculated the relative importance of the attributes. Following previous research, [27] we first computed the maximum range of a coefficient accredited to each attribute. We then computed the relative importance score (expressed as a percentage) of that particular attribute as its maximum range divided by the sum of all attribute ranges. The relative importance score reflected the relative impact of the considered attribute on the total utility a participant could receive from legal or illegal cannabis. The scores summed to 100%.

Mixed logit estimation and relative importance calculation were first conducted in the overall sample then in subgroups by cannabis use purposes and frequency. The subgroup analysis may inform heterogeneities in associations and policymaking in target populations.

We also predicted market shares of legal and illegal cannabis by estimating the number of users in each market with policy simulations in the overall sample. [29] Specifically, we predicted percentages of chosen alternatives from the mixed logit model with different hypothetical policies imposed on legal cannabis. A benchmark policy scenario was created with a few assumptions (Technical Note S2). The benchmark policy scenario was not meant to reflect the complicated real world, but to provide a reference for the comparison between different policies. The following policy scenarios were simulated: (1) a policy requiring legal cannabis with low quality improved to medium quality, (2) a policy requiring legal cannabis with low or medium quality improved to high quality, (3) a policy requiring lab test for all legal cannabis, (4) a policy allowing delivery services such that 1/2 legal dispensaries provide delivery services, (5) a policy increasing dispensary density such that dispensaries that were located 50 miles away now are located 10 miles away, (6) a policy banning dispensaries within 1 mile from residential areas, (7) a policy restricting all legal cannabis to 10% THC level, (8) a policy imposing a 10% sales tax on legal cannabis, (9) a policy imposing a 20% sales tax on legal cannabis, and (10) a policy imposing a 30% sales tax on legal cannabis.

All analyses were conducted using Stata (Version IC 16.1).

## Results

### Sample characteristics

Table S1 reports descriptive statistics of study sample and compares them to the 2019 NSDUH. Overall, the demographic characteristics were comparable.

### Mixed logit model results

Table 2 shows coefficient estimations for the overall sample.

**Table 2:** Mixed Logit Regression results: associations between Choice attributes and preferences for legal and illegal Cannabis

Attribute	Legal	Illegal	Legal vs. Illegal
	Coefficient (Standard Error)		Wald Test Statistic (*P* value)
**Alternative Specific Constant (Reference: Opt-out)**	0.65** (0.20)	0.78*** (0.23)	0.24 (*P* = 0.62)
**Quality**			
Low	*Reference*	*Reference*	NA
Medium	1.27*** (0.11)	0.81*** (0.13)	6.68** (*P* = 0.0097)
High	2.01*** (0.15)	1.27*** (0.13)	18.48*** (*P* = 0.000017)
**Lab Test**			
No	*Reference*	NA	NA
Yes	0.67*** (0.089)		
**Distance between Seller and Home**			
Deliverable	1.95*** (0.14)	1.69*** (0.15)	2.17 (*P* = 0.14)
1 mile	1.94*** (0.14)	1.45*** (0.14)	7.78** (*P* = 0.0053)
10 miles	1.41*** (0.15)	1.34*** (0.15)	0.13 (*P* = 0.72)
50 miles	*Reference*	*Reference*	NA
**THC Level**			
Low: 10%	-0.45*** (0.13)	NA	NA
Medium: 20%	0.12 (0.12)		
High: 30%	0.36** (0.12)		
Unknown	*Reference*		
**Price**	-0.031*** (0.0053)	-0.087*** (0.0074)	30.98*** (*P* = 2.60 × 10^− 8^)
Number of Choices	26,001		
Number of Participants	963		

*Notes*: 1. *p < 0.05, **p < 0.01, ***p < 0.001. THC stands for tetrahydrocannabinol

2. This table estimates a model for the probability of choosing an alternative over others. The “legal” column shows coefficients on attribute levels and a constant interacted with a legal alternative indicator; the “illegal” column shows those interacted with an illegal alternative indicator

3. The null hypothesis of a Wald test is that the corresponding coefficients for legal and illegal cannabis are equal. Under the null hypothesis, the Wald test statistic follows a Chi-square distribution with one degree of freedom

All the coefficients were statistically significant and in expected directions. Cannabis consumers had greater preferences for cannabis that had a greater quality, closer proximity to the seller, and a lower price regardless of the legality status. Two attributes were unique to legal cannabis: lab test and THC level. In terms of lab test, cannabis consumers preferred legal cannabis that was lab tested. In terms of THC level, cannabis consumers preferred legal cannabis with, from most to least: high, medium, unknown, and low THC.

For the three attributes that were common to both legal and illegal cannabis, there were heterogeneities in preferences between legal and illegal cannabis. Cannabis consumers had a stronger preference for legal cannabis when legal and illegal cannabis had the same quality and other attributes were held constant (Wald tests *Ps* < 0.01). The coefficient of price for illegal cannabis was greater than that for legal cannabis (Wald test *P* < 0.01), indicating that preferences for illegal cannabis were more price-sensitive. Transforming the coefficients to price elasticities, a 10% price increase in legal cannabis would reduce the choice probability of legal cannabis by 2.3%, whereas the same price change in illegal cannabis would reduce the choice probability of illegal cannabis by 4.4%.

Tables 3 and 4 report coefficient estimates by cannabis use purposes and frequency, respectively. The overall preference patterns persisted in subgroup analysis. Some interesting observations are noteworthy. Among medical-only users, for illegal cannabis a shorter distance was not always preferred. Similar observations were also seen among recreational users and occasional users regarding accessibility.

**Table 3:** Mixed Logit Regression results: associations between Choice attributes and preferences for legal and illegal Cannabis. By Cannabis Use purposes

Attribute	Medical User		Recreational User		Dual User	
	Legal	Illegal	Legal	Illegal	Legal	Illegal
	Coefficient (Standard Error)					
**Alternative Specific Constant (Reference: Opt-out)**	-0.42 (0.50)	0.49 (0.78)	0.68* (0.34)	0.97** (0.34)	1.07*** (0.32)	0.64 (0.37)
**Quality**						
Low	*Reference*	*Reference*	*Reference*	*Reference*	*Reference*	*Reference*
Medium	1.07* (0.53)	0.19 (0.51)	1.40*** (0.20)	0.86*** (0.20)	1.22*** (0.16)	0.90*** (0.18)
High	2.00*** (0.45)	0.72 (0.65)	1.95*** (0.22)	1.28*** (0.20)	2.04*** (0.20)	1.46*** (0.20)
**Lab Test**						
No	*Reference*		*Reference*		*Reference*	NA
Yes	1.01** (0.35)		1.01*** (0.16)		0.36** (0.12)	
**Distance between Seller and Home**						
Deliverable	2.00*** (0.38)	1.51* (0.75)	2.20*** (0.25)	1.67*** (0.26)	1.89*** (0.19)	2.03*** (0.22)
1 mile	1.82*** (0.33)	0.69 (1.28)	2.26*** (0.24)	1.96*** (0.25)	1.76*** (0.19)	1.61*** (0.21)
10 miles	1.39*** (0.31)	1.62*** (0.40)	1.68*** (0.23)	1.44*** (0.25)	1.34*** (0.19)	1.46*** (0.21)
50 miles	*Reference*	*Reference*	*Reference*	*Reference*	*Reference*	*Reference*
**THC Level**						
Low: 10%	0.015 (0.45)		-0.34 (0.21)		-0.66*** (0.18)	NA
Medium: 20%	0.49 (0.34)		0.22 (0.20)		0.0047 (0.19)	
High: 30%	0.58 (0.61)		0.37 (0.21)		0.43* (0.18)	
Unknown	*Reference*		*Reference*		*Reference*	
**Price**	-0.0018 (0.014)	-0.100*** (0.020)	-0.043*** (0.0085)	-0.096*** (0.012)	-0.028** (0.0086)	-0.081*** (0.015)
Number of Choices	4698		10,422		10,881	
Number of Participants	174		386		403	

*Notes*: *p < 0.05, **p < 0.01, ***p < 0.001. THC stands for tetrahydrocannabinol

**Table 4:** Mixed Logit Regression results: associations between Choice attributes and preferences for legal and illegal Cannabis. By Cannabis Use frequency

Attribute	Occasional User		Regular User	
	Legal	Illegal	Legal	Illegal
	Coefficient (Standard Error)			
**Alternative Specific Constant (Reference: Opt-out)**	0.30 (0.26)	0.56 (0.31)	1.33*** (0.34)	1.25*** (0.35)
**Quality**				
Low	*Reference*	*Reference*	*Reference*	*Reference*
Medium	1.24*** (0.13)	0.60*** (0.17)	1.33*** (0.18)	1.10*** (0.18)
High	2.02*** (0.19)	1.07*** (0.19)	2.08*** (0.23)	1.54*** (0.18)
**Lab Test**				
No	*Reference*		*Reference*	
Yes	0.80*** (0.12)		0.51*** (0.14)	
**Distance between Seller and Home**				
Deliverable	1.77*** (0.18)	1.57*** (0.23)	2.34*** (0.24)	1.96*** (0.22)
1 mile	1.90*** (0.18)	1.39*** (0.21)	1.94*** (0.22)	1.58*** (0.21)
10 miles	1.37*** (0.16)	1.28*** (0.21)	1.68*** (0.21)	1.38*** (0.22)
50 miles	*Reference*	*Reference*	*Reference*	*Reference*
**THC Level**				
Low: 10%	-0.092 (0.18)		-0.88*** (0.18)	
Medium: 20%	0.27 (0.15)		-0.0011 (0.20)	
High: 30%	0.31* (0.14)		0.53** (0.20)	
Unknown	*Reference*		*Reference*	
**Price**	-0.025*** (0.0074)	-0.099*** (0.014)	-0.032*** (0.0080)	-0.084*** (0.0099)
Number of Choices	15,309		10,692	
Number of Participants	567		396	

*Notes*: *p < 0.05, **p < 0.01, ***p < 0.001. THC stands for tetrahydrocannabinol

### Relative importance

Figures S3 and S4 illustrate the relative importance estimates for legal and illegal cannabis, respectively. For legal cannabis, in the overall sample, cannabis consumers valued quality and accessibility (31% and 31%, respectively) more than lab test, THC level, and price (10%, 13%, and 15% respectively). Heterogeneities were observed across subgroups. For example, the importance of lab test was high among medical-only and recreational-only users (18% and 14%, respectively) but low among dual-purpose users (6%). The importance of price was extremely low among medical-only users (1%) but high among recreational-only and dual-purpose users (18% and 13%, respectively). THC level was valued much more by regular users (19%) than occasional users (7%). In contrast, lab test was valued more by occasional users (14%) than regular users (7%).

For illegal cannabis, cannabis consumers valued price (47%) more than accessibility (30%) and quality (23%). The rank orders of the relative importance for quality, accessibility, and price remained identical for all the subgroups.

### Policy simulations

Table 5 shows predictions of market shares of legal and illegal cannabis under different policy scenarios. Several policies would likely induce cannabis consumers to substitute illegal cannabis with legal cannabis, hence the market share of illegal cannabis would be decreased. Such policies included those ensuring minimum quality, requiring lab test, allowing delivery services, and increasing legal dispensary density. The reduction in illegal cannabis market share ranged between 4.25% and 8.67%. In contrast, several policies would likely increase the market share of illegal cannabis. These policies included those banning dispensaries near residential areas, capping maximum THC level, and imposing sales tax. The increase in illegal cannabis market share ranged between 0.61% and 4.01%. The interpretation of these results should use caution, however, because the purpose of the policy simulation was to illustrate the trade-offs between legal and illegal markets under various hypothetical policy scenarios instead of providing precise predictions in the real world.

**Table 5:** Predicted market shares and percentage-point changes relative to the Benchmark Policy under various policy scenarios

Policy Scenario	Legal Cannabis	Illegal Cannabis	Opt-out	Legal Cannabis	Illegal Cannabis	Opt-out
	Market Share (%)			Change in Market Share Relative to Benchmark Policy (%)		
Benchmark Policy	86.4%	10.7%	3.0%	NA	NA	NA
1. A policy requiring legal cannabis with low quality improved to medium quality	94.4%	5.2%	0.4%	8.1%	-5.5%	-2.6%
2. A policy requiring legal cannabis with low or medium quality improved to high quality	98.0%	2.0%	0.0%	11.6%	-8.7%	-2.9%
3. A policy requiring lab test for all legal cannabis	93.6%	5.6%	0.8%	7.3%	-5.2%	-2.1%
4. A policy allowing delivery services such that 1/2 legal dispensaries provide delivery services	92.0%	6.5%	1.6%	5.6%	-4.3%	-1.4%
5. A policy increasing dispensary density such that dispensaries that were located 50 miles away now are located 10 miles away	94.8%	4.9%	0.3%	8.5%	-5.8%	-2.7%
6. A policy banning dispensaries within 1 mile from residential areas such that half of those dispensaries now located 10 miles away and half now located 50 miles away	80.9%	14.7%	4.4	-5.4%	4.0%	1.4%
7. A policy restricting all legal cannabis to 10% THC level	79.7%	14.7%	5.6	-6.7%	4.0%	2.7%
8. A policy imposing a 10% sales tax on legal cannabis	85.2%	11.3%	3.5%	-1.1%	0.6%	0.5%
9. A policy imposing a 20% sales tax on legal cannabis	84.1%	12.1%	3.9%	-2.3%	1.4%	0.9%
10. A policy imposing a 30% sales tax on legal cannabis	83.0%	12.6%	4.4%	-3.4%	1.9%	1.4%

## Discussion

This study suggested that cannabis quality, safety, accessibility, potency, and price were all associated with cannabis consumers’ preferences for cannabis flower regardless of the legality status (note that safety and potency were not assessed for illegal cannabis). This finding is supported by previous literature using or not using an experimental approach. For example, a DCE study among medical cannabis consumers with chronic pain found that cannabis quality was an important factor in cannabis selection. [30] A purchase task study indicated that perceived quality influenced cannabis demand, with users being willing to pay more for a higher quality. [31] A recent systematic review summarized observational studies and reported a consistent, positive association between cannabis availability/accessibility and cannabis use. [32] Another DCE study found that cannabis consumers preferred higher THC but cannabis nonusers did not. [33] The same DCE study along with the two purchase tasks conducted by Amlung et al. all reported that a lower price was associated with a greater likelihood of choosing cannabis or a greater consumption of cannabis. [12, 13, 33]

Cannabis consumers seemed to value the same attribute level change differently between legal and illegal cannabis. For example, they attached a greater utility to an increase in quality level for legal cannabis (from low to medium, or from medium to high) than illegal cannabis. Cannabis consumers were also more price-sensitive to illegal cannabis than legal cannabis. These observations concur with Amlung et al. [12, 13], who concluded that illegal cannabis was considered inferior to legal cannabis by cannabis consumers.

Heterogeneities by cannabis use purposes and frequency were revealed in relative importance calculations for legal cannabis. Medical-only users attached a much lower importance to price but a higher importance to quality and safety than recreational-only and dual users. Patients have a more stable demand for cannabis to treat chronic conditions, such that price may play a less important role in their purchase decisions. Product quality may be more important to medical-only users as reliable sources of cannabis are needed to constantly treat their conditions whereas contaminations may exacerbate existing conditions or even lead to new ones. Regular users weighed potency much more important than occasional users, possibly because they are more tolerant or addicted to cannabis. These heterogeneities suggested that cannabis policies may generate differential impacts on these subgroups and specific policies can be designed to target a certain subgroup. For example, lowering legal cannabis prices is more likely to encourage the shift from illegal to legal markets among recreational users while improving legal product quality and safety is more likely to motivate the shift among medical-only users. Similarly, regular users may be more responsive to THC regulations in legal markets than occasional users.

Policy simulations provided recommendations if reducing the size of illegal cannabis markets is the primary policy goal. It should be acknowledged that, however, the spillover effects of legal cannabis regulation on illegal markets should not be the only consideration in cannabis policymaking. The increasing share of legal cannabis markets may be also undesired, due to public health concerns associated with the increase in problem cannabis use and related health and social consequences. With this caveat in mind, our study suggested that policies improving product quality, ensuring safety, allowing delivery services, and increasing dispensaries licenses may have potential to increase legal cannabis market share and reduce illegal cannabis market share. In contrast, policies banning dispensaries in residential areas, restricting THC to a low level, and imposing sales taxes may unintendedly heighten the harms from illegal cannabis. Because empirical evidence on cannabis policy impacts is lacking, existing U.S. cannabis policies were mainly drawn from the lessons and success of tobacco control in the U.S., where illegal markets are not a major source for purchase. Policymakers are encouraged to take a holistic view considering public health, social, and economic consequences of cannabis regulations in both legal and illegal markets.

Our study has limitations. First, this study is not free from limitations that are common to all DCE studies. For example, participants’ responses may have deviated from their real-life decisions due to the hypothetical nature of the experiment. Previous research showed that DCEs reasonably predicted real-world choices. [34, 35] We also attempted to mitigate the bias by educating participants before the experiment and emphasizing the importance of the research to science and policymaking. DCEs also simplify real-life decisions by restricting the number of choices and attributes, which is unfortunately inevitable for reducing cognitive burden and inferring causality from experimental research. Moreover, product attributes were standardized and known in our DCE but in the real world consumers may have different understanding particularly in illegal markets.

Second, this study focused on cannabis flower to standardize choices in legal and illegal markets. Nonetheless, alternative forms of cannabis such as concentrates and edibles have been rising in market shares. [36] Future research is warranted to investigate consumer choices on these alternative forms.

Further, we did not consider other sources of obtaining cannabis (e.g., unlicensed dispensaries, home cultivation) in the DCE.

Lastly, we used quota matching to make the sample representative of the U.S. adult cannabis consumer population, but the generalizability of the findings may be still limited due to convenience sampling approach. Our results cannot generalize to cannabis nonusers or youths in the U.S. or other countries that have different legal contexts.

## Conclusion

In the U.S., cannabis consumers’ preferences for illegal cannabis were associated with both legal and illegal cannabis product attributes. Policies regulating legal cannabis markets should consider potential spillover effects to illegal markets.

## Electronic supplementary material

Below is the link to the electronic supplementary material.

Supplementary Material 1

Supplementary Material 2

## Data Availability

The datasets generated and/or analyzed during the current study are not publicly available due to the sensitive data on drug use but are available from the corresponding author on reasonable request.

## Abbreviations

THC: tetrahydrocannabinol
DCE: Discrete choice experiment
NSDUH: National Survey on Drug Use and Health
