# Supplementary material for: Cannabis consumers’ preferences for legal and illegal cannabis: evidence from a discrete choice experiment
Source: BMC Public Health. 2024 Sep 4;24:2397. doi: 10.1186/s12889-024-19640-1 (PMC11373389; doi:10.1186/s12889-024-19640-1)
Supplement: Supplementary file 1 — Supplementary Material 1 [file 12889_2024_19640_MOESM1_ESM.pdf]

Survey S1: Questionnaire Excerpt for the Discrete Choice Experiment

Demographics

In what state do you currently live?

- O California (1)
- O Colorado (2)
- O Massachusetts (3)
- O Maine (4)
- O Michigan (5)
- O Nevada (6)
- O Oregon (7)
- O Washington (8)
- O Other (9)

What is your age?

O Less than 18 (1)

O 18-20 (2)

O 21-24 (3)

O 25-29 (4)

O 30-34 (5)

O 35-39 (6)

O 40-44 (7)

O 45-49 (8)

O 50-54 (9)

O 55-59 (10)

O 60-64 (11)

O 65-69 (12)

O More than 70 (13)

What is your sex?

O Male (1)

O Female (2)

What is the highest grade you completed?

- O Less than 11th grade (1)
- O 12th grade, no diploma (2)
- O High school graduate - high school diploma or the equivalent (GED) (3)
- O Some college, no degree (4)
- O Associate degree (5)
- O Bachelor's degree (6)
- O Master's degree (7)
- O Professional or Doctorate degree (8)

Are you Hispanic, Latino/a, or Spanish origin?

- O Yes (1)
- O No (2)

Which category below best describes your race?

- O White (1)
- O Black or African American (2)
- O Asian (3)
- O Other (4)

Marijuana Use Frequency and Purposes

Marijuana is also called weed, herb, pot, grass, bud, ganja, or Mary Jane. Marijuana can be smoked, vaped, and mixed with edibles and drinks.

In the past 12 months, have you used marijuana at least once?

- O Yes (1)
- O No (2)

In the past 12 months, on how many days did you use marijuana regardless of methods using it? Please enter a number in any one of the boxes below. You can respond using whatever method is easiest for you to recall.

- O Average number of days **PER WEEK** during the past 12 months (1) ____
- O Average number of days **PER MONTH** during the past 12 months (2) ____
- O Approximate **TOTAL** number of days during the past 12 months (3) ____

In the past 30 days, on how many days did you use marijuana regardless of methods using it?

In the past 12 months, did you use marijuana primarily for medical purposes to treat or decrease symptoms for a health condition, or primarily for recreational purposes to get pleasure or satisfaction?

- O Primarily for medical purposes (1)
- O Primarily for recreational purposes (2)
- O For both medical and recreational purposes (3)

Discrete Choice Experiment Question

PLEASE READ CAREFULLY!
Although your state has legalized medical and recreational marijuana, illegal market for marijuana still exists. The next set of questions are about choices you may make when you consider where to buy marijuana flowers.

We would like you to choose between 2 sources of marijuana flowers:

- A **legal** dispensary, which is either a medical marijuana dispensary or a recreational marijuana retail dispensary selling you marijuana legally.
- An **illegal** dealer, who is a person selling you marijuana illegally. A dealer includes strangers but also friends and relatives who may sell you marijuana.

PLEASE READ CAREFULLY!
Marijuana purchased from a legal dispensary or an illegal dealer can be described by several characteristics. Below we describe 5 characteristics, for each of which we vary by different ""levels".

1. Marijuana Quality.

- <u>Low-quality</u> marijuana flowers are very dry, full of seeds and stems, and more brown than green.
- <u>Medium-quality</u> marijuana flowers can be identified by their spectrum of green hues and the presence of colorful pistils. Seeds and stems are minimal, but the flowers can suffer from a number of quick-to-market techniques like improper flushing of nutrients, quick curing methods, and sloppy trim jobs.
- <u>High quality</u> marijuana flowers are primarily green in color with a brilliant array of vibrant colors. Seeds and stems are extremely rare. The flowers themselves are typically dense and chunky.

2. Lab Tested. Marijuana flowers sold in legal dispensaries are often tested by a third-party testing lab for residual pesticides, unwanted contaminants, and the presence of mycotoxins like mold and mildew. Flowers sold by illegal dealers are not tested.

3. Distance to Home. Distance of legal dispensary or illegal dealer to your home will be described by number of miles. Dispensaries or dealers may also deliver marijuana to your home directly.

4. THC Level. THC is the primary psychoactive ingredient in marijuana to get you high. There is also some evidence suggesting that THC provides certain health benefits. Dried flowers tend to range between 10% and 30% THC. Marijuana products bought from a legal dispensary

usually come with labels THC labels revealing THC level, but THC level for flowers bought from an illegal dealer is unknown.

5. Price. Please assume you would like to buy 1/8 ounce marijuana flowers, which are roughly equivalent to 3.5 grams or 5 joints. The prices that you see in this survey may not be the prices that you pay in reality. This is ok; please use the prices that we provide when you consider the choices. Assume that the price you see is the full cost we would like you to consider in making your choice.

PLEASE READ CAREFULLY!

In addition to the characteristics mentioned above, there are other characteristics of legal dispensary and illegal dealer. We assume that the following characteristics are constant in all the choices:

Marijuana from a Legal Dispensary	Marijuana from an Illegal Dealer
Compliant with the law	Against the law
No criminal penalties apply for purchase	Criminal penalties apply for purchase
There are a variety of strains for you to choose from	There are limited number of strains for you to choose from
Known manufacturing or cultivation facility that provides the products	Unknown sources

Below is an **EXAMPLE** of a choice question (you DO NOT need to click anything). A person selected illegal dealer from the 2 options.

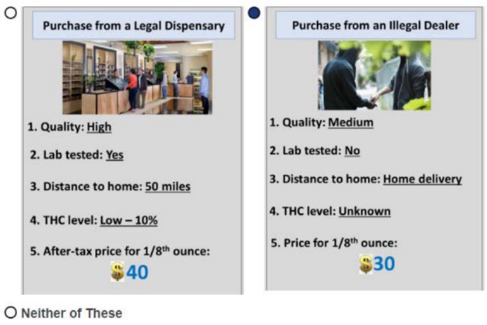

On the next few pages, you will be asked to purchase marijuana from **2 sources (legal dispensary vs. illegal dealer) 9 different times**. Note that:

- Assume the 2 sources are your ONLY options.
- You can select "neither of these" if you do not want to buy marijuana or do not want to buy from these 2 sources.
- The 2 sources will have different combinations of characteristic levels in each question. Please consider ALL of the characteristics when you make a choice.
- Remember that illegal dealer could be not only strangers but also friends and relatives.
- Select the source of marijuana you like better based on its characteristic levels; it is okay if you selected a different source in another question.

There are no right or wrong answers.
Your choices will provide very useful information for a research study.
Thank you for your time and effort!!

Below is an example of a discrete choice experiment question (a total of 81 questions were grouped into 9 blocks and each participant answered one block of 9 questions).

Instructions:

- Assume the 2 sources are your ONLY options.
- You can select "neither of these" if you do not want to buy marijuana or do not want to buy from these 2 sources.
- The 2 sources will have different combinations of characteristic levels in each question. Please consider ALL of the characteristics when you make a choice.
- Remember that illegal dealer could be not only strangers but also friends and relatives.
- Select the source of marijuana you like better based on its characteristic levels; it is okay if you selected a different source in another question.
- There are no right or wrong answers.

Task 1/9

Imagine that you would like to buy marijuana flowers. Would you like to buy from a legal dispensary or an illegal dealer?

○ **Purchase from a Legal Dispensary**

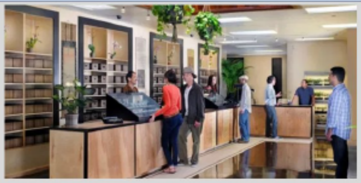

1. **Quality:** <u>Medium</u>
2. **Lab tested:** <u>Yes</u>
3. **Distance to home:** <u>Home delivery</u>
4. **THC level:** <u>Unknown</u>
5. **After-tax price for 1/8th ounce:**

$50

○ **Purchase from an Illegal Dealer**

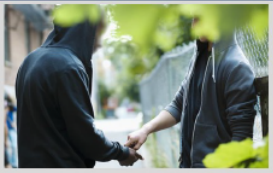

1. **Quality:** <u>Medium</u>
2. **Lab tested:** <u>No</u>
3. **Distance to home:** <u>50 miles</u>
4. **THC level:** <u>Unknown</u>
5. **Price for 1/8th ounce:**

$20

○ Neither of These
