# Supplementary material for: Cannabis consumers’ preferences for legal and illegal cannabis: evidence from a discrete choice experiment
Source: BMC Public Health. 2024 Sep 4;24:2397. doi: 10.1186/s12889-024-19640-1 (PMC11373389; doi:10.1186/s12889-024-19640-1)
Supplement: Supplementary file 2 — Supplementary Material 2 [file 12889_2024_19640_MOESM2_ESM.docx]

**SUPPORTING INFORMATION**

**Figure S1: Example of a Discrete Choice Experiment Choice Scenario**


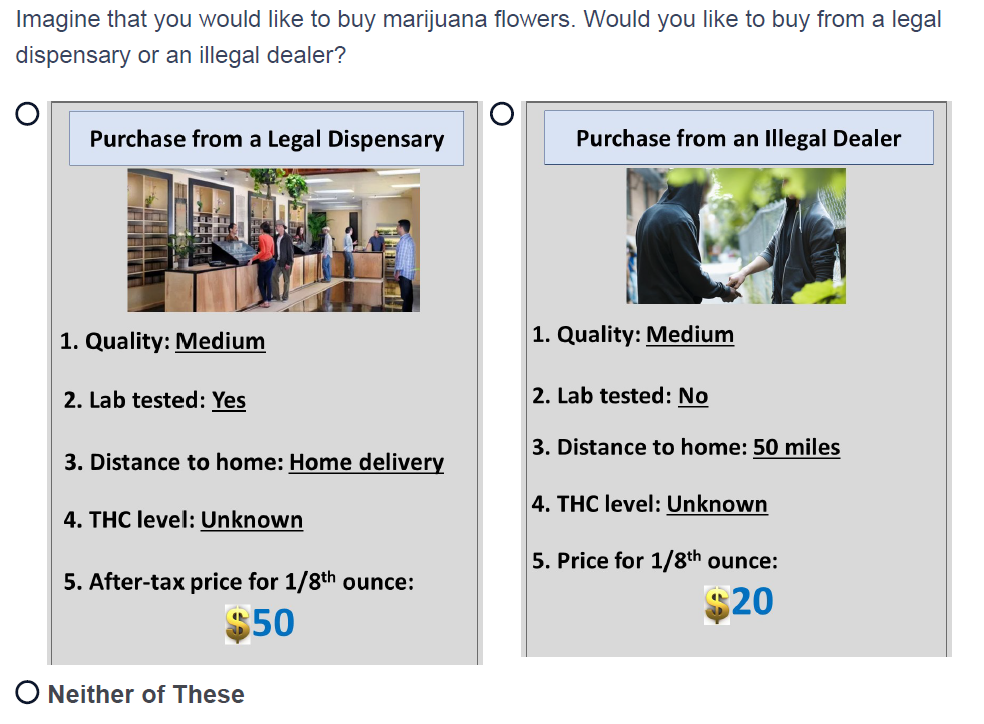


Notes: THC stands for Tetrahydrocannabinol.

Technical Note S1. Definitions of Cannabis Quality Levels

- Low-quality marijuana flowers are very dry, full of seeds and stems, and more brown than green.


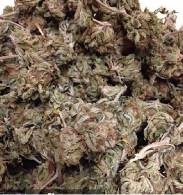


- Medium-quality marijuana flowers can be identified by their spectrum of green hues and the presence of colorful pistils. Seeds and stems are minimal, but the flowers can suffer from a number of quick-to-market techniques like improper flushing of nutrients, quick curing methods, and sloppy trim jobs.


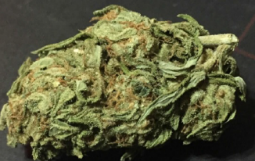


- High quality marijuana flowers are primarily green in color with a brilliant array of vibrant colors. Seeds and stems are extremely rare. The flowers themselves are typically dense and chunky.


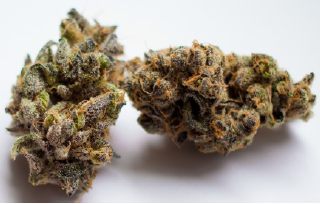


**Figure S2. Factors Not Described by the DCE Attributes**


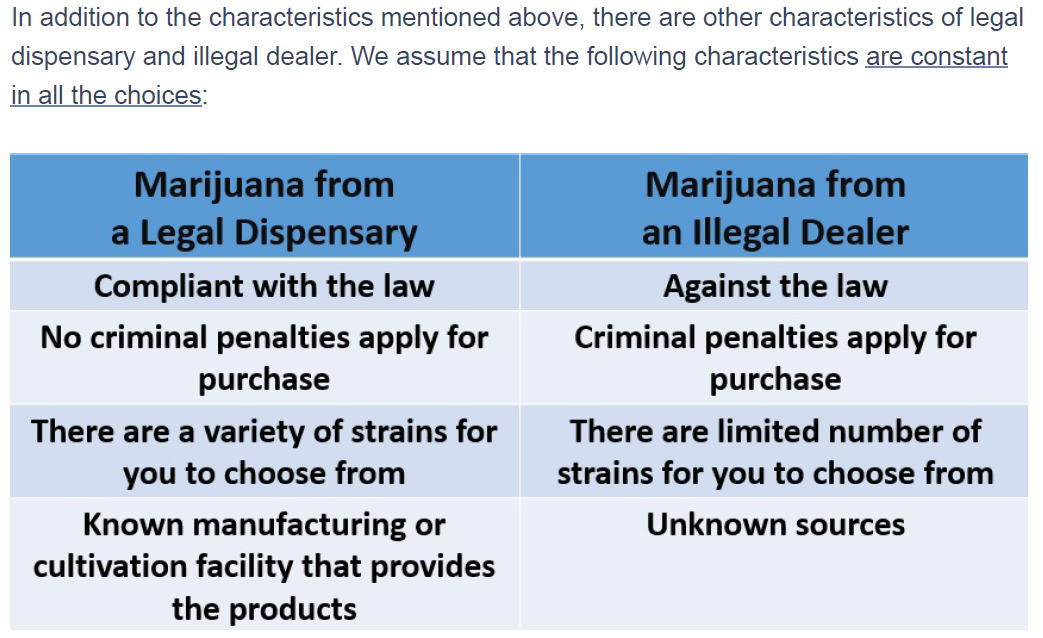


**Technical Note S2. Assumptions in the Benchmark Policy Scenario**

1. For both legal and illegal cannabis, low, medium, and high qualities are evenly distributed (1/3 each).
2. For both legal and illegal cannabis, lab test is not present.
3. For both legal and illegal cannabis, equal number of sellers are located 1-, 10-, and 50-mile away (1/3 each). Deliveries are not allowed.
4. For legal cannabis, 10%, 20%, 30%, and unknown THC levels are evenly distributed (1/4 each). All illegal cannabis has unknown THC level.
5. For both legal and illegal cannabis, $20, $30, $40, and $50 prices are evenly distributed (1/4 each).

**Table S1. Study Sample Characteristics and Comparison to 2019 National Survey on Drug Use and Health (NSDUH)**

| **Variable** | **Study Sample** | **2019 NSDUH** |
| --- | --- | --- |
|  | **%** | |
| Age |  |  |
| 21-34 | 50.26 | 46.95 |
| 35+ | 49.74 | 53.05 |
| Sex |  |  |
| Male | 58.57 | 57.70 |
| Female | 41.43 | 42.30 |
| Educational Attainment |  |  |
| High School Degree or Less | 33.02 | 31.50 |
| Some College/Associate Degree or College Graduate | 66.98 | 68.50 |
| Race and Ethnicity |  |  |
| Hispanic | 14.33 | 13.32 |
| Non-Hispanic White | 70.72 | 67.06 |
| Non-Hispanic Black | 8.00 | 13.24 |
| Non-Hispanic Other Minorities | 6.96 | 6.38 |
| Cannabis Use Purposes |  |  |
| Medical-only | 18.07 | NA |
| Recreational-only | 40.08 | NA |
| Dual-purpose | 41.85 | NA |
| Cannabis Use Frequency |  |  |
| Occasional Use | 58.88 | 70.08 |
| Regular Use | 41.12 | 29.92 |
|  |  |  |
| Number of Participants | 963 | 8,405 |

Notes:

1. The analysis of 2019 NSDUH was restricted to past-year cannabis users.
2. The statistics of cannabis use purposes are not reported for NSDUH because its definitions are not comparable with our study.

**Figure S3: Relative Importance of Attributes for Legal Cannabis**

| 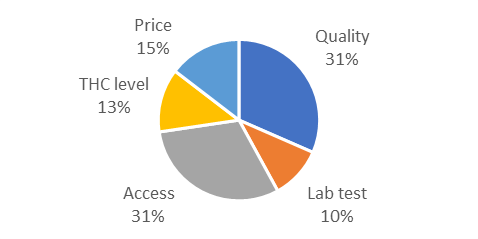   1. All | 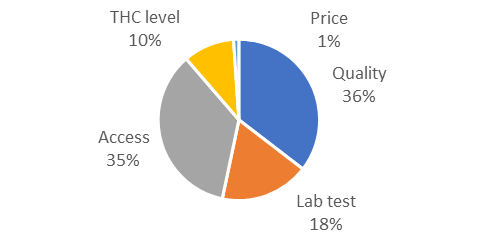   1. Medical-only User |
| --- | --- |
|  |  |
| 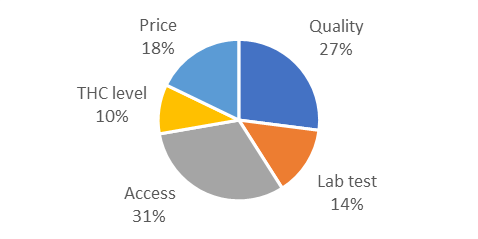   1. Recreational-only User | 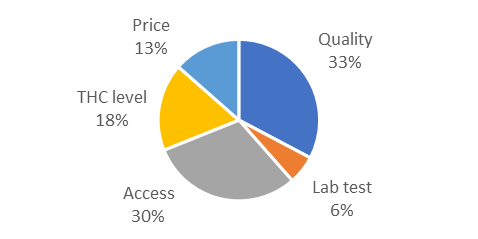   1. Dual-purpose User |
| 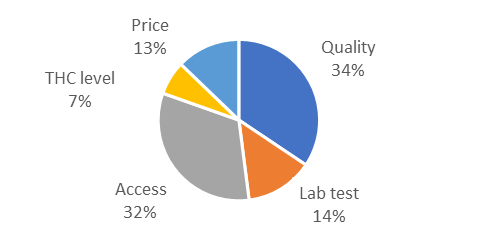   1. Occasional User | 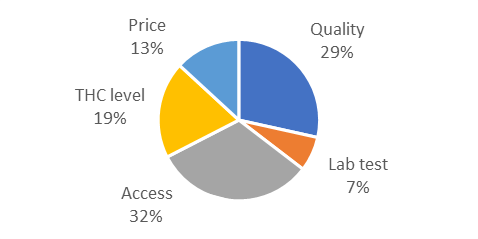   1. Regular User |

Notes: THC stands for Tetrahydrocannabinol.

**Figure S4: Relative Importance of Attributes for Illegal Cannabis**

| 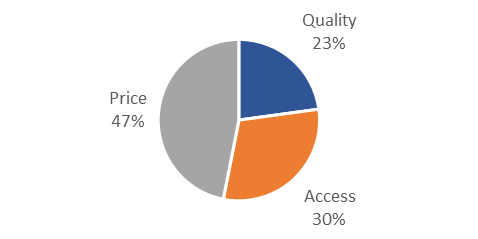   1. All | 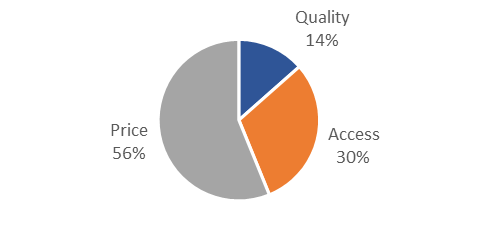   1. Medical-only User |
| --- | --- |
|  |  |
| 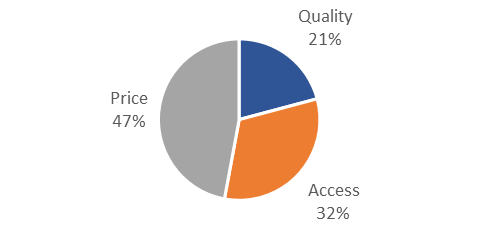   1. Recreational-only User | 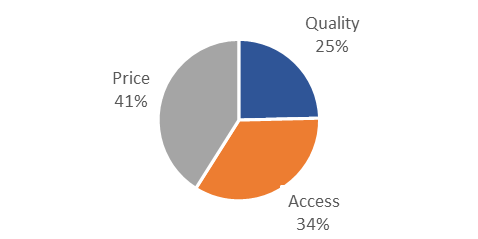   1. Dual-purpose User |
| 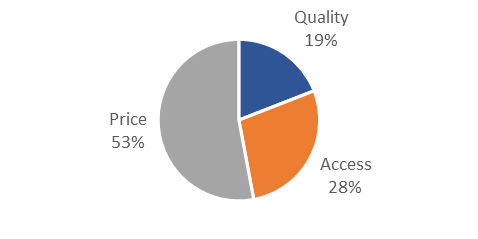   1. Occasional User | 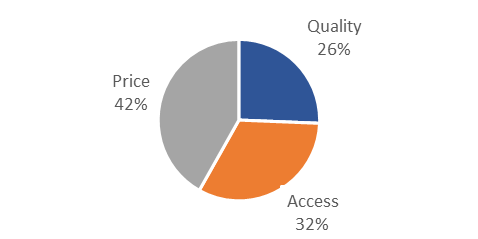   1. Regular User |

Notes: THC stands for Tetrahydrocannabinol.
